# Role of complement in myasthenia gravis

**DOI:** 10.3389/fneur.2023.1277596

**Published:** 2023-10-05

**Authors:** Pyae Phyo San, Saiju Jacob

**Affiliations:** ^1^Institute of Immunology and Immunotherapy, University of Birmingham, Birmingham, United Kingdom; ^2^Department of Neurology, Queen Elizabeth Hospital Birmingham, University Hospitals Birmingham NHS Foundation Trust, Birmingham, United Kingdom

**Keywords:** myasthenia gravis, AChR antibody, complement, C5, eculizumab, ravulizumab, zilucoplan, meningococcal

## Abstract

Myasthenia gravis is a prototypic neuroimmune disorder with autoantibodies targeting the acetylcholine receptor complex at the neuromuscular junction. Patients present with mainly ocular muscle weakness and tend to have a generalized muscle weakness later in the clinical course. The weakness can be severe and fatal when bulbar muscles are heavily involved. Acetylcholine receptor antibodies are present in the majority of patients and are of IgG1 and IgG3 subtypes which can activate the complement system. The complement involvement plays a major role in the neuromuscular junction damage and the supporting evidence in the literature is described in this article. Complement therapies were initially studied and approved for paroxysmal nocturnal hemoglobinuria and in the past decade, those have also been studied in myasthenia gravis. The currently available randomized control trial and real-world data on the efficacy and safety of the approved and investigational complement therapies are summarized in this review.

## 1. Introduction

Myasthenia gravis (MG) is a neuroimmunological disorder where the autoantibodies target the nicotinic acetylcholine receptor (AChR) complex at the postsynaptic membrane of the neuromuscular junction (NMJ) of various skeletal muscles.

The incidence varies from 1.7–21.3 per million person-years for all myasthenia types and 4.3 to 18 per million person-years for AChR MG and an estimated United Kingdom (UK) prevalence of 15 per 100,000 population (1, 2).

Clinical presentation arises from the fatigability of various skeletal muscles. At the onset, it is limited to extraocular muscles in about 85% of patients, giving rise to symptoms such as diplopia, blurred vision, and ptosis. The muscles involved will become generalized in about 80% of such patients, mainly within 2 years from onset (3). Neck, limb, bulbar, and respiratory muscles can be involved with various presentations such as head drop, dysarthria, dysphagia, dyspnoea, and limb weakness. About 40% of patients have severe muscle weakness involving the bulbar and respiratory muscles. One in five patients with severe muscle weakness require ventilator support with endotracheal intubation. With the lack of such ventilation assistance in the past, respiratory failure and pneumonia used to be the causes of almost 100% mortality in the earlier centuries. Despite the advances in ventilator support, mortality remains around 5% to 10% (3).

MG is a prototypic T-cell dependent B-cell mediated autoimmune disorder and anti-AChR antibody is elevated in 90% of patients with generalized MG and 50% with localized ocular MG (3). Muscle specific kinase (MuSK) antibody is found to be positive in about 70% of AChR antibody-negative patients (4). In about 8% of double seronegative patients, low-density lipoprotein receptor-related protein 4 (LRP4) antibody is positive (5–7).

Among the different antibodies identified in myasthenia gravis, AChR antibody is of IgG1 and IgG3 subtype and can activate the complement system. In this article, we will only review AChR-MG with the focus on the role of the complement system in the pathogenesis and its therapeutic potential.

## 2. Role of complement in AChR-MG

### 2.1. Proposed pathogenic mechanisms of AChR antibody

AChR is of the larger ligand-gated ion channel gene superfamily and the best-known nicotinic AChR of the family. It is a transmembrane glycoprotein structure and composed of five homologous subunits α2βγδ as fetal AChR, and in the adult type, the γ subunit is replaced by the ε subunit.

The AChR is a very potent immunogen (8). The ability to induce experimental autoimmune MG in several animal models either actively by heterologous or homologous AChR or its parts or passively by polyclonal or monoclonal AChR antibodies has been shown in several studies (9, 10).

Over half of the autoantibodies were observed to bind to the α subunit of AChR, especially to the major immunogenic region (MIR) formed by overlapping epitopes in the Extracellular domain of the α subunit (α 67–76). Autoantibodies can bind all AChR subunits, including the γ subunit in fetal AChR. However, α subunit binding antibodies were found to be more pathogenic (8, 11, 12).

**Figure 1:**
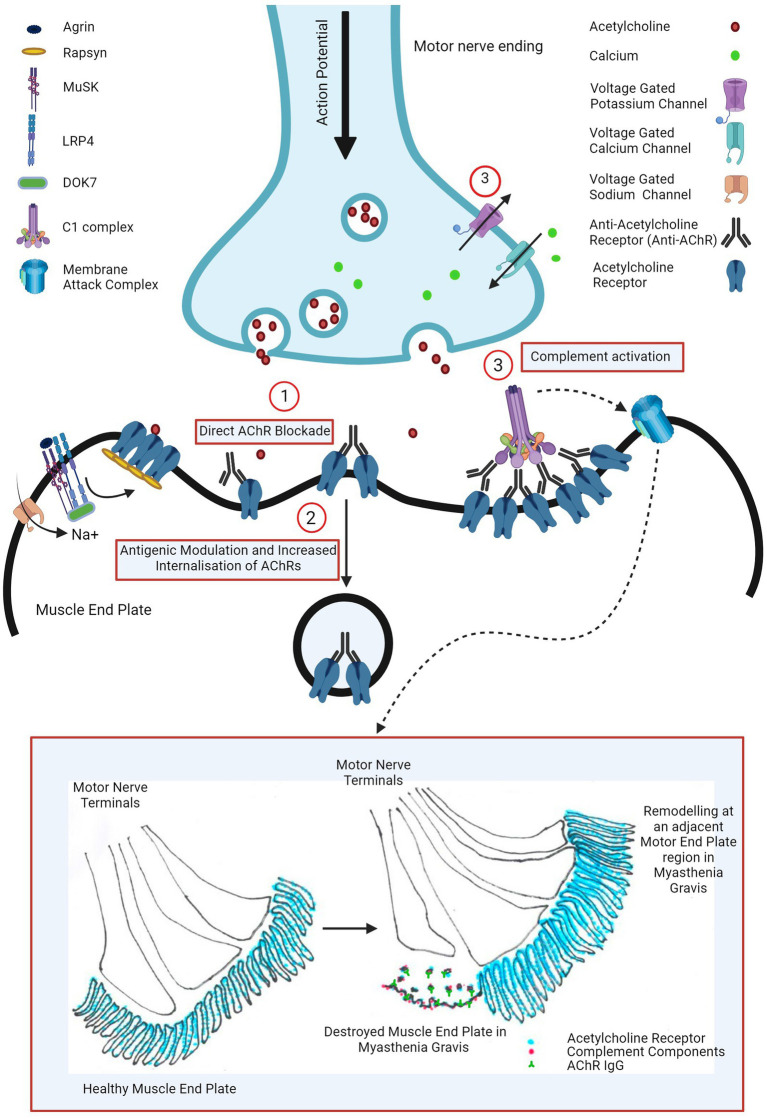
Pathogenic mechanisms of AChR antibody in myasthenia gravis: (1) direct AChR blockade, (2) antigenic modulation and increased AChR internalization, and (3) complement activation leading to complement mediated NMJ destruction (widening of primary synaptic cleft (space between motor nerve terminal and muscle end plate), destruction of junctional folds with simplification of secondary clefts (fewer and wider clefts), increased AChR, AChR IgG and complement bound junctional fold debris in the primary synaptic cleft) and remodeling of nearby motor end plate region (motor nerve terminal moving away from the damaged muscle end plate area with formation of a new end plate in a nearby region). Image created with the help of ^©^BioRender.com.

Three pathogenic mechanisms of AChR antibodies have been proposed in the literature and are schematically presented in Figure 1.

The first proposed mechanism is the direct AChR blockade, where anti-AChR binds to and directly inhibits AChR function. Various groups have explored this hypothesis using human, mice and rat cells, transfected cell lines and intact neuromuscular junctions however such studies failed to show a unanimous neurotransmission failure (13). At the physiological NMJ, AChRs are densely packed at around 9,000 receptors per square micrometre (14) and perhaps the results may have been consistent if this had been taken into consideration. Cetin et al. (13) exploited this and demonstrated that by mimicking a physiological NMJ by clustering AChRs using rapsyn in CN21 cell line, AChR antibody sera from patients were able to rapidly and more potently block AChR currents than in the cell line with unclustered AChRs.

However, the magnitude of AChR antibody bound to AChR at the post-synaptic membrane was observed to be directly proportionate to the AChR index (residual AChR) and the mini-end plate potential in the muscle biopsies from myasthenic patients. This suggested that direct antibody blockade may not be the most important mechanism and the receptor depletion mechanisms may play a larger role for neurotransmission failure (15).

The AChR population at the post synaptic membrane is rather dynamic due to internalization and either recycling or degradation and replacement with new receptors (16). Engel at al demonstrated that this process was accelerated in EAMG rats compared with healthy control ones however this was also compensated by increased synthesis and release of AChR in mild or subclinical EAMG rats (15). Heinemann et al. (17) demonstrated similar findings in rat diaphragm using anti-AChR rat sera in rat diaphragm and Dranchman et al. (18) by patient-derived immunoglobulins in rat muscle cell cultures. This antigenic modulation by anti-AChR antibody seems to be mediated by its receptor cross-linking ability (19, 20).

In most severely affected EAMG mice, junctional folds were however destroyed and shedding of labelled AChR into the synaptic space was seen and Engel et al. inferred that this cannot by explained purely by an increased AChR internalization mechanism (15).

In the muscle biopsies of myasthenic patients, similar ultrastructural changes were also observed such as widening of primary synaptic clefts with significant debris of junctional folds in the synaptic clefts, simplification of junctional folds (by shallowing and widening or reduction in the number of secondary synaptic clefts), remodeling of the endplate and moving away of the nerve terminal from the destroyed endplate to an adjacent region where a new endplate region was formed (illustrated in Figure 1) (15, 21, 22). Such NMJ loss suggested complement involvement in the pathogenesis of myasthenia gravis.

### 2.2. Complement in myasthenia gravis

Complement system is part of innate immune response and central functions include inducing acute inflammation, killing microbes by opsonization for phagocytosis and osmotic/colloidal lysis and removing apoptotic host cells. It can help solubilize or remove antigen-antibody complexes from circulation. It is also involved in adaptive immune response by helping regulate T and B cell activation (23–25).

It is an integrated system of nearly 50 proteins present abundantly in blood but not in normal extravascular tissues. Complement is activated on cell surfaces of mainly microbes and damaged host cells and autoimmunity is suppressed by complement regulators present at the intact cell surfaces (see below for a further review in relation to MG pathogenesis). It operates in a cascade via a series of proteolytic cleavages after activation. IgM and IgG are major immunoglobulins that can activate the complement cascade via the classical pathway. IgG can diffuse into normal extravascular tissues. In contrast IgM can only enter those with increased vascular permeability induced by tissue inflammation.

There are three pathways to activate the complement cascade however only the classical pathway is the most relevant in this review, and it will be described.

#### 2.2.1. The classical pathway

**Figure 2:**
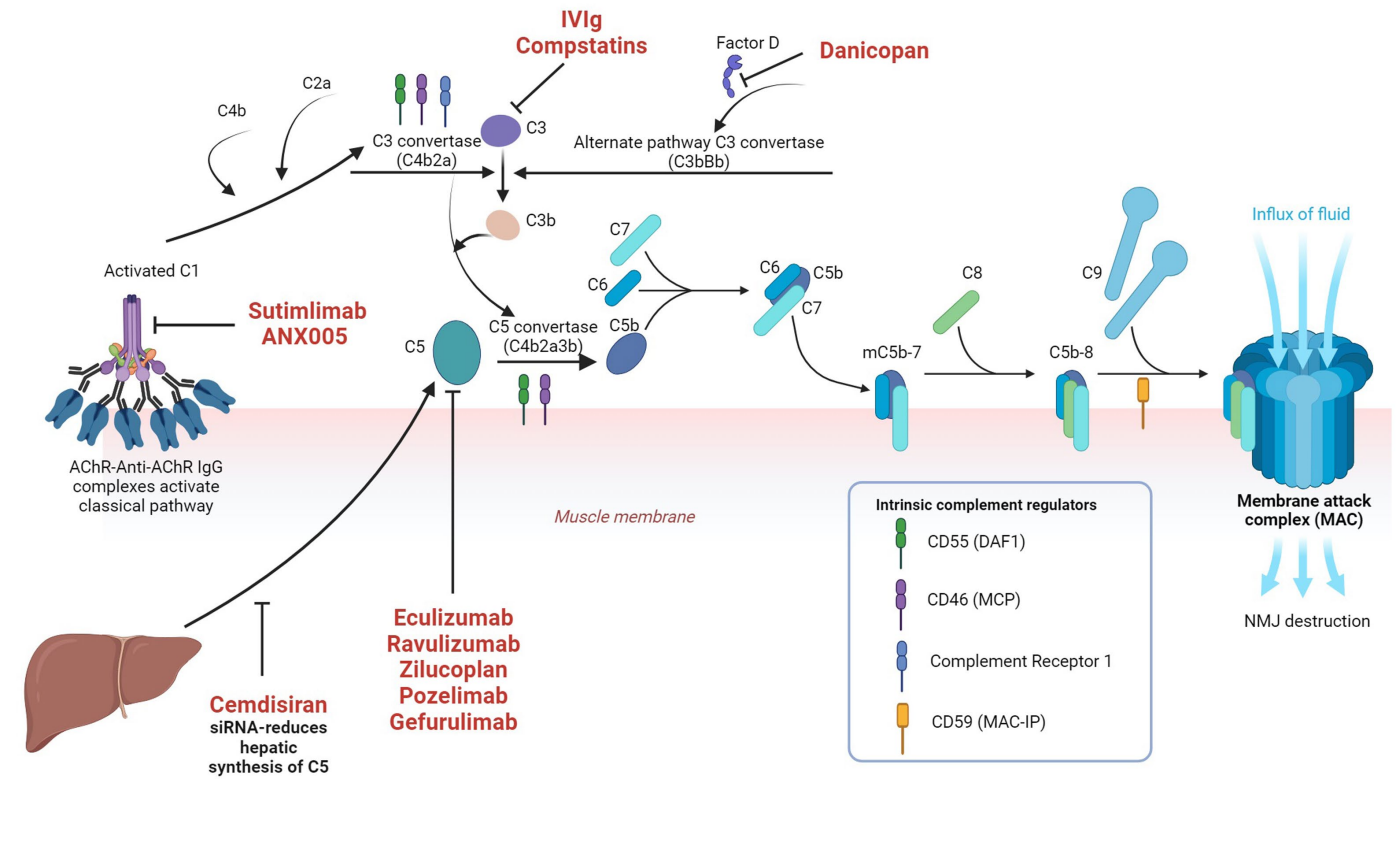
Classical complement pathway in myasthenia gravis and the approved and investigational therapeutic targets in the complement system Image created with the help of ^©^BioRender.com.

Multivalent C1q can either be activated by direct binding to microbes or by antigen antibody complexes and subsequent enzymatically active C1r and C1s are generated. C1s cleaves C4 to C4a and C4b. C1r, C1s and C4b in combination cleaves C2 to C2a and C2b. C4b2a complex (C3 convertase) cleaves C3 to C3a and C3b. C3C4b2a3b forms C5 convertase and initiates terminal complement pathway by cleaving C5 into C5a and C5b. C3a, C4a and 5a are anaphylatoxins, which are proinflammatory and responsible for increased vascular permeability, smooth muscle contraction and leucocyte recruitment. C5b subsequently exposes a binding site for C6 and C5bC6 reversibly binds to the cell surface and forms the foundation for membrane attack complex (MAC). C7 binds to C5bC6 to form C5bC6C7, which subsequently induces transmembrane insertion of C8α and C8β, forming unstable pores. C9 binds to C8α and attracts polymerization of multiple C9 molecules to stabilize the pores with a maximum diameter of 10 nm. This C5bC6C7C8C9 forms MAC, which lyses the cell via several mechanisms (25). The classical pathway is illustrated in Figure 2 alongside the approved and investigational therapeutic targets.

#### 2.2.2. Telltale of NMJ destruction by the complement system

The possibility of the complement system involvement in the pathogenesis of myasthenia was first considered in 1960s when sera from myasthenic patients were able to cause cytolytic destruction of frog sartorius muscle fibers correlating with serum complement levels outside a normal range in most patients (26).

Studies have demonstrated that antibodies binding AChR leads to complement deposition at the NMJ (27, 28). In the muscle biopsies of patients with myasthenia, IgG and C3 deposition were localized at the identical sites such as the post-synaptic membrane, synaptic cleft debris and on disintegrating junctional folds suggesting that the complement pathway had been activated by anti-AChR and it had been completed to C3 phase (15). C9 is one of the major components in assembling the final and stable membrane attack complex, which is responsible for destruction of neuromuscular junction in case of myasthenia gravis. As definitive evidence of destruction of NMJ by the complement system, Engel et al. (15) demonstrated that deposition of C9 was seen at the MG end plate regions however the most intense depositions were observed in association with the most abnormal looking and destroyed neuromuscular junctions. Such findings were also reflected in EAMG models (29).

In myasthenic patients with high AChR antibody concentrations, the evidence of consumption of complement was also observed and C3 levels were inversely correlated with clinical severity in AChR-MG patients (30, 31). The electrophysiological tests often correlate with *in vitro* serum complement-fixing ability of clustered AChR ab (32).

The essential role that the complement plays in the pathogenesis of AChR-MG was also supported by prevention of murine EAMG either by depleting the complement with cobra venom factor or by knocking out C3, C4, C5 or C6 (33–36). In all of these studies, despite visualization of antibodies attached to AChR at NMJ, neither complement deposition nor NMJ destruction were observed.

#### 2.2.3. How anti-AChR IgG could trigger complement cascade

In the classical pathway, IgM, a natural pentamer in blood, when bound to an antigen, can strongly active the complement cascade. However, it is not relevant to AChR-MG as the most specific antibody is IgG. Unlike the pentameric IgM, IgGs in oligomers, can bind C1q with a sufficient avidity to activate the complement system (37, 38). Among IgG subclasses, IgG 1 and 3 are known to be the strongest complement activators.

Six IgG monomers can form a hexamer via Fc:Fc interactions and are able to bind and activate C1q and subsequently the downstream complete cascade (39–41). For lateral recruitment model on a sparsely antigenic surface, IgG must be monovalent to be able to form hexamers as bivalent binding seems to suppress oligomerization by lateral collision, however with vertical recruitment from the solution, IgGs can be bivalent (41). For high density antigenic expression like nicotinic NMJ AChRs, the former model is not important (41). This has been illustrated in a few studies but not in myasthenia. Such Fc:Fc interactions can be modified by Fc mutations or Fc domain deglycosylation (39, 40).

AChR antibodies are of bivalent IgG1 and 3 subtype and can cross-link the receptors with antibodies against the major immunogenic region being the most potent (11). It can be deducted that densely populated AChRs with an estimated distance of a mere 10 nm between them favour a very close clustering of Anti-AChR monomers, formation of hexamers via the vertical pathway mentioned above and thereby a multivalent binding of C1q multivalent head. However, the direct visualizable evidence of how the complement cascade is triggered by AChR IgGs in AChR-MG is yet to be investigated.

#### 2.2.4. Complement regulators

Complement regulators are present at host cell surfaces to prevent autologous destruction by the complement system (42). Decay accelerating factor 1 (DAF1) or CD55 inhibits C3 and C5 convertases and accelerates their decay (43). Membrane cofactor protein (CD46) is a cofactor of cleavage of C3b and C4b, which form C3/4 convertases (44). Complement receptor 1 accelerates the decay of C3/5 convertases and degradation of C3b/4b (45). Membrane attack complex inhibitory protein (MAC-IP) or CD59 inhibits the formation of membrane attack complex (46, 47).

Mice that are deficient in DAF1 alone or both DAF1 and CD59 were observed to be much susceptible to EAMG with significant receptor loss, muscle weakness and NMJ damage, with the double knockout mice showing a significantly worse EAMG even leading to crisis (48, 49).

Complement regulator activities (mRNA and protein expression) were observed to be lower at extraocular muscles (EOM) than diaphragm at baseline or after EAMG induction in mice. This supports increased predilection of EOM involvement in AChR-MG patients (50).

#### 2.2.5. Complement system as therapeutic targets

##### 2.2.5.1. High dose intravenous immunoglobulin

Among the established immunomodulatory therapies for MG, intravenous immunoglobulin (IVIg) appears to inhibit the complement system by neutralization of C3a and C5a and at high concentrations, by inhibiting the uptake of C3b and C5b onto the cell surface and subsequent complement mediated tissue damage (51, 52).

##### 2.2.5.2. FDA approved anti-C5 therapies

###### 2.2.5.2.1. Eculizumab

Eculizumab is a recombinant humanized monoclonal antibody against C5. It binds to C5 and prevents its breakdown to C5a and C5b, thereby reducing inflammatory cells recruitment and membrane attack complex formation. United States Food and Drug Administration (U.S. FDA) first approved its use in paroxysmal nocturnal hemoglobinuria (PNH) in 2007 and atypical haemolytic uremic syndrome (aHUS) in 2011, where uncontrolled complement activation is largely responsible for the pathogeneses.

It was then studied in 2 neurological conditions such as myasthenia gravis and aquaporin-4 (Aqp-4) antibody positive neuromyelitis optica spectrum disorder (NMOSD), where complement involvement is seen but less well-delineated. Phase 3 randomized controlled trial (RCT) data for Aqp-4-positive NMOSD (PREVENT, prevention of relapses in neuromyelitis optica; NCT01892345) showed a remarkable 94% relative risk reduction of relapses (53) however the benefits were less clear in the RCT data for MG.

It is the first complement therapy investigated in MG. Fourteen patients with severe refractory generalized MG (gMG) were studied for 16 weeks with a crossover in a phase 2 randomized, double blind placebo-controlled trial. A clinically meaningful response was produced with 86% achieving primary end point of three-point reduction in QMG score and a significant overall QMG score reduction between treatment and placebo group (*p* = 0.0144) (54).

Encouraging results from phase 2 RCT led to a multinational, randomized, placebo-controlled, double-blind phase 3 study (REGAIN, safety and efficacy of eculizumab in AChR positive refractory generalized myasthenia gravis; NCT01997229) in a similar population as the phase 2 study (55). One hundred twenty-five AChR-antibody positive refractory severe generalized MG patients from North America, Latin America, Europe and Asia were enrolled into the study. Eligibility criteria were myasthenia gravis-activities of daily living (MG-ADL) score of 6 or more, myasthenia gravis foundation of America (MGFA) class II–IV disease, vaccination against *Neisseria meningitides*, and previous treatment with at least two immunosuppressive therapies (IST) or one immunosuppressive therapy and chronic IVIg or plasma exchange (PLEX) for 12 months without symptom control. Exclusion criteria were history of thymoma or thymic neoplasms, thymectomy within 12 months before screening, or use of IVIg or PLEX within 4 weeks before randomization, or rituximab within 6 months before screening. Patients had either intravenous (IV) eculizumab or placebo 900 mg on day 1, weeks 1, 2 and 3 and 1,200 mg in week 4 for induction phase, and thereafter maintenance dose of 1,200 mg every 2 weeks up to 26 weeks.

Primary endpoint was the change in MG-ADL score from baseline to week 26 using worst-rank ANCOVA (analysis of covariance) and REGAIN failed to reach a statistical significance (*p* = 0.0698). It was likely attributed by the use of worst-rank analysis that assigned patients who discontinued eculizumab regardless of the reason to the lowest ranks. Three out of seven patients who discontinued eculizumab due to adverse events rather than worsening of myasthenia were given the lowest ranks despite a clinically meaningful benefit. However, pre-specified secondary efficacy endpoints showed statistically significant benefits. The changes in quantitative myasthenia gravis (QMG) and myasthenia gravis quality of life 15-item scale (MGQoL-15) scores using worst-rank ANCOVA from the baseline met statistical significances at *p* = 0.0129 and *p* = 0.0281 but the changes in myasthenia gravis composite (MGC) scores did not. Prespecified responder analyses of MG-ADL and QMG showed that eculizumab group had a higher proportion of patients with a clinically meaningful improvement than the placebo group. In prespecified sensitivity analysis of all 4 scores (MG-ADL, QMG, MGQoL-15 and MGC), treatment group had significantly lower scores than the placebo group, which were sustained throughout from week 1 to 26. Hence the authors inferred that the use of derived rank rather than the actual change in the scores affected the primary end point outcome negatively and all the evidence from both primary and secondary endpoint analyses should be considered in the interpretation of REGAIN trial outcome (55).

The commonest adverse events identified were headache, upper respiratory infection and nasopharyngitis which were of mild to moderate severity. The commonest serious adverse events were infections. There were no statistically significant differences between the treatment and placebo groups in terms of the adverse events. Complement system plays a major role in killing *Neisseria meningitidis* and hence meningococcemia is one serious risk considered with eculizumab, although no patients in REGAIN developed Meningococcal infection. Fewer patients in the eculizumab group experienced exacerbations and needed rescue therapy (55).

One hundred seventeen patients from the double-blind phase of REGAIN 56 from the blinded eculizumab group (eculizumab/eculizumab) and 61 from the blinded placebo group (placebo/eculizumab) were enrolled into the open label extension (OLE) phase for a maximum of 4 years. After a blinded induction phase (active drug provided as 1,200 mg every 2 weeks for previous eculizumab group and 900 mg on day 1 and weekly for 3 weeks for the previous placebo group), all patients were administered 1,200 mg every 2 weeks. Compared with the pre-REGAIN baseline, overall myasthenic exacerbations were reduced by 75.2% and the rate of MG related hospitalizations by over 80%. In the eculizumab/eculizumab group, the improvement in all 4 scores were sustained throughout the OLE. A rapid and significant improvement in all 4 scores was also observed in the placebo/eculizumab group from REGAIN: over 50% of improvement was seen in the first 3 months and the improvements were sustained for 30 months (56).

The safety profile from REGAIN OLE also matched with the current safety profile for generalized MG and post marketing safety profile of eculizumab in PNH and aHUS (56).

A retrospective analysis by Howard et al. (57) looked at the responder subgroup from RCT and OLE phases of REGAIN. Early and late responders were defined by clinically meaningful improvements in MG-ADL reduction by ≥3 points from the baseline or QMG reduction by ≥5 points from the baseline before week 12 or after week 12. 67.3% and 56.1% from eculizumab group of RCT phase were identified as early responders by MG-ADL and QMG improvements, respectively, but with longer duration of the treatment, more responders were increasingly identified. The response to eculizumab treatment was sustained until the end of the OLE as indicated by 84.7% and 71.4% responder proportion in MG-ADL and QMG.

Another post-hoc analysis of REGAIN looked at the proportion of patients who attained minimal symptom expression (MSE) as defined by MG-ADL scores 0–1 or MGQoL-15 scores 0–3. A significantly higher number of patients in the eculizumab group achieved MSE at week 26 of REGAIN than in the placebo group [MG-ADL: 21.4% vs. 1.7%; 95% confidence interval (CI) 8.5, 31.0; *p* = 0.0007; MGQoL-15: 16.1% vs. 1.7%; 95% CI 4.3, 24.6; *p* = 0.0069] (58). At week 130 of OLE phase, the proportion of patients with MSE in placebo/eculizumab group significantly increased (MG-ADL: 1.7 to 27.8%; MG-QoL15: 1.7 to 19.4%) and in eculizumab/eculizumab group, MSE was maintained at similar proportions to the RCT phase (58).

The response of immunosuppressive therapies in myasthenic patients are assessed with MGFA post intervention status (MGFA-PIS) and the consensus therapeutic efficacy goal is aimed at MGFA-PIS minimal manifestations (MM), which is defined as having no symptoms of functional limitations but with some weakness on examination or better. At week 26 of REGAIN RCT phase, a higher proportion of eculizumab group achieved better MGFA-PIS than the placebo group [improved (60.7% vs. 41.7%) or MM (25.0% vs. 13.3%); common OR: 2.3; 95% CI: 1.1–4.5]. In the OLE at week 130, 88% achieved improved status and 57.3%, MM status (59).

A small number of patients needed regular IVIg use for at least 12 months (17 patients) or rituximab (14 patients) prior to REGAIN and constituted the extreme spectrum of refractory MG, not dissimilar to real-world clinical practice (60, 61). A sufficient washout period of at least 4 weeks for IVIg and 6 months for rituximab was given before enrollment into REGAIN, to minimize bias.

IVIg subgroup had a higher exacerbation rate at pre-REGAIN baseline than the overall REGAIN cohort (150.0 versus 102.4 exacerbations/100 patient-years). At week 26 of REAGIN RCT phase, eculizumab treated group attained a clinically meaningful response (reduction of MG-ADL scores ≥3 points and QMG scores ≥5 points) in 75% compared with only one-fifth (in terms of QMG) to one-third (in terms of MG-ADL) improvement in the placebo group. Such improvements were also sustained in eculizumab/eculizumab group at 71% at the interim analysis at week 52 during OLE phase. Placebo/eculizumab group showed a rapid and sustained improvement at OLE phase. Compared with pre-REGAIN baseline, at 18 months, overall hospitalization rate was reduced by 68% however it was not statistically significant due to a small sample size of this subgroup. This data supported durable benefits of eculizumab in this sub-cohort (60).

In the rituximab subgroup in REGAIN, a significantly higher proportion had an exposure to ≥4 ISTs, which could reflect a super-refractory nature of this sub-cohort with the possibility of an increased cumulative risk from several ISTs. However, the safety profile and the efficacy of eculizumab use in this subgroup were comparable to the non-rituximab group (61).

A rapid and significant clinical improvement with the use of eculizumab was also reported in a case series of ventilator-dependent AChR-MG patients, who had been refractory to 3 or 4 immunotherapies. Two patients achieved MGFA-PIS MM within 4 to 6 weeks from the initiation of eculizumab with a sustained improvement. The third patient had a slow and partial but sustained improvement, and he was able to remain on intermittent non-invasive ventilation at week 40 of eculizumab therapy (62). Severe bulbar weakness in a refractory myasthenic crisis rapidly improved as early as 1 week with a complete resolution of bulbar symptoms within 10 weeks of eculizumab initiation (63).

The benefits and tolerability of eculizumab from REGAIN was also reflected in the evidence from real-world studies (64, 65). Fifteen treatment-refractory AChR-MG patients treated with eculizumab in a real-world study showed that a clinically meaningful reduction of mean MG-ADL score was seen as early as 3 months with further reduction at 6 and 12 months. Mean exacerbations per patient per year was reduced by 2.33 from the baseline. Burden from concomitant use of ISTs was also reduced with use of eculizumab: mean prednisolone dose was reduced by 23.33 mg/day and all 6 patients on IVIg were able to wean off from IVIg successfully. Nine out of fifteen patients also discontinued pyridostigmine at 12 months of eculizumab therapy (65).

Rituximab is a monoclonal anti-CD20 B-cell depletion therapy, and it is considered in a multi-IST refractory AChR-MG in a case-by-case basis as the response rates were not as consistent as in anti-MuSK MG. Nelke et al. (65) compared the responses in 57 MG patients treated with rituximab with those in 20 with eculizumab in a real-world retrospective 24 months observational study. A better treatment outcome was associated with eculizumab than with rituximab in terms of QMG score reduction from the baseline and MGFA-PIS MM state were more frequently achieved by the eculizumab cohort, although the risk of myasthenic crisis did not differ in both groups.

Successful uses of eculizumab in seronegative, paediatric and thymoma associated MG patients have also been reported (66–69).

REGAIN (RCT, OLE and various post-hoc subgroup analyses), real-world data and case reports mentioned above have consistently shown the rapid and sustained clinical improvement by eculizumab but its role as a first-line agent and duration of therapy are yet to be investigated with further studies. Eculizumab is currently approved for use in generalized AChR-MG (USA, 2017), refractory AChR-MG (EU) and AChR-MG unresponsive to IVIg/PLEX (Japan). Currently, it remains one of the most expensive medicines in the world and an estimated annual cost of over half a million U.S. dollars has thus been a major hindrance for a wider use (70).

###### 2.2.5.2.2. Ravulizumab

Ravulizumab is another recombinant human monoclonal antibody against C5 with a similar mechanism of action to eculizumab but with a longer half-life hence intravenous infusion are less frequent for maintenance (8 weekly as opposed to 2 weekly for eculizumab).

In phase 3 CHAMPION-MG randomized, placebo-controlled study (NCT03920293), 175 patients with AChR antibody positive gMG were randomly assigned 1:1 to receive ravulizumab versus placebo. A single loading dose on day one was administered at weight-dependent dosage of 2,400 mg for ≥40 to <60 kg, 2,700 mg for ≥60 to <100 kg and 3,000 mg for ≥100 kg, followed by maintenance doses of 3,000 mg, 3,300 mg and 3,600 mg respectively, every 8 weeks starting from day 15. The primary endpoint of significant mean change in MG-ADL in treatment vs. placebo groups was achieved (−3.1 vs. −1.4; *p* < 0.001) (71). A significantly higher proportion in the treatment group than in the placebo group was observed to attain a clinically meaningful response as defined by reduction of QMG scores by ≥5 points (30.0% vs. 11.3%, *p* = 0.005) (71). A rapid improvement was seen within 1 week of treatment initiation in ravulizumab group (71). Adverse events rates were comparable between the two groups. The open label extension phase of this study up to 60 weeks showed a sustained improvement (72). U.S. FDA approved Ravulizumab for use in MG in April 2022.

##### 2.2.5.3. Other anti-C5 therapies, which have just finished phase 3 clinical trial or on ongoing phase 3 trial

###### 2.2.5.3.1. Zilucoplan

Zilucoplan, a subcutaneously (SC) administered small macrocyclic peptide is another terminal complement inhibitor acting via two mechanisms. Its binding to C5 prevents its cleavage and the binding to the existing C5b prevents C5b’s attachment to C6. The advantages of zilucoplan are (1) patients can self-administer and thus it can be more convenient, (2) a good NMJ penetration is more likely due to its small size and (3) as it is not an antibody like eculizumab or ravulizumab, it can be co-administered either with IVIg or neonatal Fc receptor (FcRN) inhibitors (73).

Safety and efficacy of zilucoplan in patients with generalized myasthenia gravis (RAISE; NCT04115293) is a multinational randomized placebo-controlled phase 3 trial. One hundred seventy-four patients with anti-AChR positive gMG were assigned 1:1 to treatment and placebo groups and participants self-administered either zilucoplan 0.3 mg/kg or matched placebo once daily for 12 weeks. Primary efficacy endpoint was met with a significant improvement in the change of MG-ADL scores from the baseline to week 12 in zilucoplan group compared with the placebo group [least squares mean change −4·39 (95% CI −5·28 to −3·50) vs. −2·30 (−3·17 to −1·43); least squares mean difference −2·09 (−3·24 to −0·95); *p* = 0·0004] (74). Treatment emergent adverse events (TEAEs) were comparable in both groups and the commonest TEAE was injection site bruising (74). An OLE phase is currently ongoing.

###### 2.2.5.3.2. Pozelimab and/or cemdisiran

Pozelimab is a fully humanized IgG4 monoclonal antibody targeting C5 and cemdisiran is a small interfering ribonucleic acid (siRNA) which interferes with mRNA for C5 and decreases its hepatic synthesis and hence the circulating level of C5. With a loading dose of 15 mg/kg IV followed by four SC doses of 400 mg once weekly, pozelimab was able to inhibit complement activation in healthy volunteers (75). Co-administration of pozelimab with cemdisiran allowed lower and less frequent doses as compared to individual agents given separately in animal studies (76). A phase 3 randomized controlled trial of the combination (intravenous pozelimab loading followed by 4 weekly subcutaneous injections along with cemdisiran subcutaneous 4 weekly) versus placebo in generalized MG is ongoing (NCT05070858).

##### 2.2.5.4. Future investigational complement inhibition therapies

Currently, several newer therapies are being investigated at different phases of clinical trials for MG and other complement mediated disorders. These include ANX005 (anti-C1q), sutimlimab (anti-C1s), cinryze, berinert and ruconest (C1-esterase inhibitors), compstatins (a group of cyclic peptides, which bind and interfere with the function of C3), tesidolumab, crovalimab, zimura, gefurulimab and nomacopan (all target C5), SKY59 (anti-C5 as well as FcRn inhibitor), avacopan (anti-C5aR1) and danicopan (anti-factor D) (77, 78). Avacopan and danicopan are orally administered. A summary of the main complement therapies in MG is given in Table 1.

**Table 1:** Complement therapies: U.S. FDA approved or on ongoing clinical trials for myasthenia gravi.

Drug	Specific targets in the complement system	Studied group	Regimen	RCT evidence
Eculizumab	Recombinant humanized IgG2/4 monoclonal antibody against C5	AChR+ gMG	IV; induction of 900 mg weekly for 4 weeks followed by 1,200 mg maintenance every 2 weeks	Phase 3 RCT results: QMG: eculizumab vs. placebo = 54.7 vs. 70.7 (*p* = 0.0129); MG-QoL-15: eculizumab vs. placebo = 55.5 vs. 69.7 (*p* = 0.0281) (REGAIN, NCT01997229) U.S. FDA approved for treatment of adults with AChR+ gMG
Ravulizumab	Long-acting recombinant humanized monoclonal antibody against C5	AChR+ gMG	IV; weight-based dose. A single loading dose of 2,400–3,000 mg followed by maintenance doses of 3,000–3,600 mg every 8 weeks	Phase 3 RCT results: MG-ADL from baseline in treatment vs. placebo = −3.1 vs. −1.4 (*p* < 0.001) (CHAMPION MG, NCT03920293) U.S. FDA approved for treatment of adults with AChR+ gMG
Zilucoplan	Macrocyclic peptide binding C5 and C5b	AChR+ gMG	SC; once daily dose of 0.3 mg/kg	Phase 3 study showed positive results. MG-ADL from baseline in treatment vs. placebo = least squares mean change −4·39 (95% CI −5·28 to −3·50) vs. −2·30 (−3·17 to −1·43); least squares mean difference −2·09 (−3·24 to −0·95) (*p* = 0·0004) (RAISE, NCT04115293)
Pozelimab	Fully humanized IgG4 monoclonal antibody inhibiting C5 complement	AChR+ or LRP4+ gMG	SC; alone or in combination with cemdisiran	Phase 3 study is ongoing (NCT05070858)
Cemdisiran	siRNA suppressing hepatic C5 synthesis	AChR+ or LRP4+ gMG	SC; alone or in combination with pozelimab	Phase 3 study is ongoing (NCT05070858)
Gefurulimab (ALXN1720)	Anti-C5 humanized bi-specific VHH antibody (nanobody)	AChR+ gMG	SC; weight-based dose once weekly	Phase 3 study is ongoing (NCT05556096)
Danicopan (ALXN2050)	Small molecule complement pathway factor D inhibitor	AChR + gMG	Oral; 120 mg or 180 mg	Phase 2 study is ongoing (NCT05218096)

AChR, acetylcholine receptor; MG, myasthenia gravis; MG-ADL, myasthenia gravis activities of daily living score; MGC, myasthenia gravis composite score; MG-QoL-15, myasthenia gravis quality of life 15-item scale; gMG, generalized myasthenia gravis; LRP-4, low-density lipoprotein receptor related protein 4.

#### 2.2.6. Safety of complement therapies

Eculizumab is the first U.S. FDA approved complement therapy for its use in PNH since 2007 and has been in the market for the longest duration among all complement therapies. It is generally well tolerated with the commonest side effects being headache, upper respiratory tract infections and nasopharyngitis. Membrane Attack Complex is primarily responsible for killing gram negative bacteria especially *Neisseria* species and the use of eculizumab is associated with 1,000 to 2,000 times increased risk of meningococcal disease (79). Thus U.S. FDA approved prescribing information includes a boxed warning with regards to an increased risk of meningococcal infection in patients on eculizumab (79). Meningococcal vaccinations are recommended at least 2 weeks before eculizumab is initiated. The Advisory Committee on Immunization Practices (ACIP) recommends that eculizumab recipients receive both quadrivalent meningococcal conjugate (MenACWY) and serogroup B (MenB) meningococcal vaccines (80). Despite prior meningococcal vaccination, there have been reports of invasive and even fatal meningococcal diseases in patients on eculizumab therapy (81–83). Thus anti-microbial prophylaxis is also recommended by clinicians and public health agencies while the patient is on eculizumab therapy and for 3 months after discontinuation (81). However, neither vaccination nor anti-microbial prophylaxis cannot eliminate the risk of severe meningococcal infections and in addition, patients may not show typical meningitis features. Thus, it is essential that health care providers and patients have a high index of suspicion for meningococcal infection. Fluoroquinolones and macrolides can block neuromuscular transmission and clinicians should avoid them to minimize the risk of myasthenic exacerbations.

Data on less than 300 pregnancy outcomes showed no increased risk of foetal malformation or foetal-neonatal toxicity. However, as a human IgG, it may cross the placenta and appear in foetal circulation. The level of eculizumab in the breast milk is undetectable or negligible. However, due to the limited data, European medicines agency recommended an individual risk benefit analysis before eculizumab is used during pregnancy or lactation (84). If complement therapy is used in children in the future, ACIP guidelines recommended additional vaccinations against *Streptococcus pneumoniae* and *Haemophilus infleunzae* type B (84). Clinically significant neutralizing antibodies have not been reported so far.

#### 2.2.7. Prediction of complement therapy responders

Unlike some conditions, validated biomarkers are currently unavailable for myasthenia gravis to assess the disease severity. Nature of the disease makes it difficult to develop such markers for myasthenia. Some investigational assays are also being developed to assess anti-AChR mediated complement activation. Obaid et al. (85) developed an assay where an HEK293T cell line with modified expression of the complement regulator genes was used to measure AChR autoantibody-mediated MAC formation through flow cytometry. Although it was rather specific, the sensitivity was not strong enough with 59.7% detection of MAC (83 out of 139 anti-AChR positive patients) and mean fluorescence intensity of MAC and clinical severity also showed a modest positive association (85). Using humanized mouse anti-AChR antibody, mouse diaphragm and normal human serum, Plomp et al. (86) were able to visualize anti-AChR driven NMJ-restricted complement damage, complement deposition at NMJ, which correlated with electrophysiological findings.

RCT data showed that not every patient who received complement therapy achieved the desirable response and hence the biomarkers either to predict or monitor the treatment response are much needed for a personalized medicine approach for cost effectiveness and minimization of unnecessary drug exposure and the related side effects in patients. Serological analysis of complement components and activation products such as C3a, C5a and soluble C5b9 and *in vitro* complement function assay such as haemolytic assays CH50 for classical pathway could reveal evidence of complement consumption, abnormal activation of the complement system and patient specific complement activation status and can be useful to monitor complement function during disease exacerbation (87). C3 levels were reported to be inversely correlated with disease severity in terms of QMG in AChR-MG patients (31). Combinations of drug levels, C5 function and complement haemolytic CH50 can be potential therapeutic monitoring assays for eculizumab in PNH and aHUS. In an aHUS study, a composite marker C3:CH50 changes significantly during induction and maintenance phases of eculizumab and correlates with disease markers (88) CH50 assay has been adopted in generalized myasthenia gravis studies. A case report described that eculizumab administration in an AChR gMG patient was able to decrease CH50 levels in line with a clinical improvement (89). CH50 assay was also used to define zilucoplan dose for optimal complement inhibition in a phase 2 trial (90). *In vitro* complement activity assays such as CH50 are haemolytic assays and the optimal assay for myasthenia gravis should be looking at the *in-vitro* direct neuromuscular junction damage by the complement. In fact, Fichtner et al. (91) showed that there was no correlation between CH50 and AChR antibody levels or disease severity in AChR antibody-positive patients. Hence, MG researchers have yet to fully explore and develop *in vitro* complement functional assays specific to MG.

Complotypes are genetic variants affecting complement activity and hence responsible for complement mediated diseases, differences between disease severities between individuals and treatment response (92). A few rare variants have been identified in PNH patients who did not respond to eculizumab. (Missense C5 heterozygous variants preventing its binding to eculizumab, HindIII polymorphism of the complement regulatory gene CR1) (92, 93).

## 3. Conclusion

We have described the available evidence of the complement system involvement in the pathogenesis of AChR-MG although the direct evidence of how complement system could be initiated specifically by anti-AChR IgGs is not available yet. We have summarized approved complement therapies backed with RCT, OLE and real-world experience data on efficacy and safety and briefly mentioned developing complement therapies in the pipeline. Biomarkers are still needed to be able to ultra-stratify MG patients into potential specific complement therapies responder group or groups so that a personalized approach could be provided to the patients in future.

## Author contributions

PS: Conceptualization, Software, Writing – original draft, Writing – review & editing. SJ: Conceptualization, Writing – original draft, Writing – review & editing.
